# Combining citizen science and molecular diagnostic methods to investigate the prevalence of *Borrelia burgdorferi* s.l. and *Borrelia miyamotoi* in tick pools across Great Britain

**DOI:** 10.3389/fmicb.2023.1126498

**Published:** 2023-04-26

**Authors:** Jinyu Shan, Ying Jia, Peter Hickenbotham, Louis Teulières, Martha R. J. Clokie

**Affiliations:** ^1^Department of Genetics and Genome Biology, University of Leicester, Leicester, United Kingdom; ^2^PhelixRD Charity, Paris, France

**Keywords:** PCR, citizen science, Lyme disease, tick, *Borrelia miyamotoi*

## Abstract

Lyme disease is the most common tick-borne disease and is caused by a group of bacteria known as *Borrelia burgdorferi sensu* lato (s.l.) complex. Sharing the same genus as *B. burgdorferi*, *Borrelia miyamotoi* is a distinct genotype that causes relapsing fever disease. This emerging tick-borne disease is increasingly becoming a concern in public health. To investigate the prevalence of *B. burgdorferi s.l.* and *B*. *miyamotoi* in ticks first, we developed a PCR (Bmer-qPCR) that targets the phage terminase large subunit (*terL*) gene carried by *B. miyamotoi*. A similar approach had been used successfully in developing Ter-qPCR for detecting *B. burgdorferi s.l.* The terL protein functions as an enzyme in packaging phage DNA. Analytical validation of the Bmer-qPCR confirmed its specificity, efficiency and sensitivity. Second, we designed a citizen science-based approach to detect 838 ticks collected from numerous sites across Great Britain. Finally, we applied Bmer-qPCR and Ter-qPCR to 153 tick pools and revealed that the prevalence of *B. burgdorferi* s.l. and *B. miyamotoi* was dependent on their geographical locations, i.e. Scotland showed a higher rate of *B. burgdorferi* s.l. and lower rate of *B. miyamotoi* carriage as compared to those of the England data. A pattern of diminishing rate of *B. miyamotoi* carriage from southern England to northern Scotland was visible. Together, the citizen science-based approach provided an estimation of the carriage rate of *B. burgdorferi s.l.* and *B. miyamotoi* in tick pools and a potential spreading pattern of *B. miyamotoi* from the south to the north of Great Britain. Our findings underscore the power of combining citizen science with the molecular diagnostic method to reveal hidden pattern of pathogen-host-environment interplay. Our approach can provide a powerful tool to elucidate the ecology of tick-borne diseases and may offer guidance for pathogen control initiatives. In an era of limited resources, monitoring pathogens requires both field and laboratory support. Citizen science approaches provide a method to empower the public for sample collection. Coupling citizen science approaches with laboratory diagnostic tests can make real-time monitoring of pathogen distribution and prevalence possible.

## Introduction

*Borrelia burgdorferi sensu* lato (s.l.) is a group of bacteria with more than 20 genospecies that have been identified. There are three dominant genospecies of *B. burgdorferi* s.l. that are implicated in causing Lyme disease (LD). *B. burgdorferi sensu stricto* (s.s.) is mainly found in the United States, whilst *B. garinii* and *B. afzelii* are present in Europe and Asia. It is crucial to emphasise that the term *‘B. burgdorferi* s.l.’ used in this manuscript refers to a group of *Borrelia* species with genomic variations, rather than a single species (Stanek and Reiter, 2011). The LD-causing bacteria are spread to humans through the bite of infected ticks. LD is the most common vector-borne disease in the US with an estimated 476,000 cases in the US annually (Kugeler et al., 2021) and >200,000 cases per year in Western Europe (Sykes and Makiello, 2017).

Spirochaete *Borrelia miyamotoi* can be transmitted by ticks and was initially discovered in ticks from Japan in 1995 (Fukunaga et al., 1995). Since then, it has been identified globally, and cases of *B. miyamotoi* infection are on the rise, with evidence of tick-borne transmission and human infections (Platonov et al., 2011; Xu et al., 2021). Tick populations are expanding globally, partly due to climate change, particularly global warming, which contributes to the growing number of tick-borne diseases (TBDs) (Gray et al., 2009).

*B. miyamotoi*, classified as a spirochete within the relapsing fever group, uniquely belongs to the Hard Tick Relapsing Fever (HTRF) category, as stated by the Centers for Disease Control and Prevention, due to its transmission by hard ticks of the genus Ixodes (Platonov et al., 2011). Although *Borrelia miyamotoi* Disease (BMD) shares many clinical features with Lyme disease (LD), BMD is more likely to produce neurological symptoms and does not typically present with the characteristic erythema migrans rash seen in LD (Krause et al., 2013). Furthermore, BMD has been associated with acute respiratory distress syndrome, which is uncommon in LD (Krause et al., 2013).

*Borrelia* species including *B. miyamotoi* have been detected in hard-bodied ticks from the genus *Ixodes*, which can be found throughout the temperate environments of the northern hemisphere. Four species act as the main vectors, *I. pacificus* and *I. scapularis* being predominant in the West and East coasts of North America respectively, whilst *I. ricinus* and *I. persulcatus* are common in Europe and Asia, respectively (Schotthoefer and Frost, 2015). The tick lifecycle, typically lasting 2 years depending on host availability, involves 3 stages: larva, nymphs and adults. At each stage, the tick feeds on a host before moulting into the next stage or reproducing if it is an adult (Murray and Shapiro, 2010). Ticks acquire *Borrelia* when feeding on infected hosts and subsequently transmit infection during future bites. Ticks feed on a range of animals, commonly small vertebrates. Some hosts, for example, the white-footed mouse act as reservoir hosts maintaining the infection and subsequently infecting many ticks, whilst others such as deer are non-permissive to infection and act to increase tick populations (Schotthoefer and Frost, 2015). Optimal conditions for ticks and reservoir hosts increase the number of infected ticks and therefore the risk of TBDs including LD (Dubrey et al., 2014).

Citizen science is an approach to empower members of the public to collaborate with research scientists for sample collection (Heigl et al., 2019). It offers a possibility of collecting many samples covering a vast geographical area, and it offers an opportunity for scientists to enhance our knowledge of TBD epidemiology. Engaging with the public is a critical aspect of citizen science, as it identifies interested citizen scientists in collecting and submitting ticks they encounter (Porter et al., 2021). The benefit of citizen science is ‘limitless’ in temporal or spatial extents. To ensure accuracy and validity of citizen science data, sampling procedure must be clearly written with fully functional collection kits provided. Information regarding sampling date, location, tick origins plus any relevant information including weather, temperature, etc. is recommended for recording. This information will help research scientists to verify the sampling procedure and data interpretation. The citizen science approach can undoubtedly provide valuable insights into TBD ecology and epidemiology. This study utilised a national citizen science-based volunteer network to investigate the *B. burgdorferi* s.l. and *B. miyamotoi* prevalence in tick pools across Great Britain.

We describe a project here that combines citizen science with PCR methods for detecting bacteria in ticks. Citizen scientists sent ticks they found from various geographic locations using our tick kit. Ticks from the same location, collected on the same date and from the same origin were pooled together. The tick pools were analysed using our newly developed Bmer-qPCR to detect *B. miyamotoi* and our previously established Ter-qPCR for *B. burgdorferi s.l.* This study revealed that the prevalence of *B. burgdorferi s.l.* and *B. miyamotoi* in tick pools in England could be as high as 67.6 and 7.1%, respectively, as compared to those of 78.8 and 4.5% in tick pools in Scotland. Overall, up to 5.2% of tick pools tested positive for both *B. miyamotoi* and *B. burgdorferi s.l.*, demonstrating presence of both *Borrelia* species in the same location. The citizen science-coupled qPCR approach can be scaled up to a broad geographic area over an extended period of time. Data generated from this study would help us understand the spread of both *Borrelia* species and how we could better manage tick populations to mitigate the risk of LD and RF from tick bites.

## Materials and methods

### Bacterial strains

The *Borrelia* strains used in this study are listed in Table 1. Professor Sven Bergström from the Department of Molecular Biology, Umea University, Sweden, provided ten strains. Seven strains were obtained from the Pasteur Institute and DSMZ (German Collection of Microorganisms and Cell Cultures GmbH). Cecilia Hizo-Teufel from the German National Reference Centre for *Borrelia* provided two strains, and two more were obtained from the Centre for Disease Control and Prevention (CDC), USA. *Borrelia* cells were cultured in 15 ml Falcon™ conical tubes containing 14 ml of Barbour-Stoenner-Kelly (BSK) II medium supplemented with 7% rabbit serum, referred to as complete BSKII or c-BSKII. The cultures were incubated at 35°C without agitation as described previously (Zuckert, 2007). The culture media were filter-sterilised using 0.22 μm pore size filters. Phase contrast microscopy (Ceti Magnum Trinocular) and a Fuchs Rosenthal Disposable Counting Chamber (C-Chip, NanoEnTek) were used for visualising and counting *Borrelia*.

**Table 1:** *Borrelia* strains used in this study.

Lab number	Isolate name	Scientific name	Source	Disease
B31	B31	*Borrelia burgdorferi*	Sven Bergström	LD
1	1,120	*Borrelia duttonii*	Sven Bergström	RF
2	Her HS1	*Borrelia hermsii*	Sven Bergström	RF
3	VS185 P9	*Borrelia burgdorferi*	Sven Bergström	LD
4	NE218	*Borrelia valasiana*	Sven Bergström	LD
5	ACA1	*Borrelia afzelii*	Sven Bergström	LD
6	UK filtered	*Borrelia burgdorferi*	Sven Bergström	LD
7	190 P9	*Borrelia garinii*	Sven Bergström	LD
8	China23	*Borrelia burgdorferi*	Sven Bergström	LD
9	CA128	*Borrelia bissettii*	Sven Bergström	LD
10	FR64b	*Borrelia miyamotoi*	CDC, USA	RF
11	HT31	*Borrelia miyamotoi*	CDC, USA	RF
12	CIP 109134	*Borrelia burgdorferi*	Institut Pasteur	LD
13	CIP 108855 T	*Borrelia spielmanii*	Institut Pasteur	LD
14	CIP 105366 T	*Borrelia lusitaniae*	Institut Pasteur	LD
15	DSM 21467	*Borrelia valasiana*	DSMZ	LD
16	DSM 10508	*Borrelia afzelii*	DSMZ	LD
17	DSM 10534	*Borrelia garinii*	DSMZ	LD
18	DSM 23469	*Borrelia bavariensis*	DSMZ	LD
19	Pbi	*Borrelia bavariensis*	Cecilia Hizo-Teufel	LD
20	NT54	*Borrelia bavariensis*	Cecilia Hizo-Teufel	LD

LD, Lyme disease; RF, Relapsing fever.

The other non-*Borrelia* strains were cultured in Clokie’s lab as part of ongoing projects. *Clostridium difficile* and *Clostridium perfringens* were cultured in brain heart infusion according to a previously established method (Shan et al., 2012). Lysogen Broth (LB) was used to culture the following bacteria: *Escherichia coli*, *Pseudomonas aeruginosa*, *Streptococcus pneumoniae*, *Staphylococcus aureus*, *Burkholderia thailandensis*, *Burkholderia pseudomallei* and *Salmonella enterica*. *Haemophilus influenzae* strains were cultured overnight at 37°C in 10 ml BHI broth supplemented with 2 μg/ml NAD and 10 μg/ml hemin (sBHI) (Turkington et al., 2019).

### Tick collection

Ethical approval for tick collection and citizen science was obtained from the University of Leicester Ethics Sub-Committee for Medicine and Biological Sciences. Ticks were collected by citizen scientists between June 2016 and October 2017.

Upon receipt from citizen scientists, adult ticks were identified using tick identification tools such as Bristol University TickID,^1^ ESCCAP UK & Ireland guidance and the UK Heath Security Agency’s ‘Be tick aware’ toolkit. Relevant information provided by the citizen scientists was recorded in a spreadsheet (Supplementary Table 1). Tick pools, which were composed exclusively of adult ticks, were stored at 4°C for up to 1 month until DNA extraction.

### DNA extraction methods

Tick pools (3–10 ticks were observed in each pool) were rinsed with 70% ethanol, followed by three washes with sterile water, and thoroughly air-dried within a class II biological cabinet. Sets of porcelain pestle and mortar were cleaned, autoclaved and used to grind ticks. During grinding, 1 ml sterile PBS was added. The homogenised grinding products were referred to as tick suspensions and were used for DNA extraction. Sterile PBS was also used as a sample in DNA extraction to create an extraction control. The DNA extraction procedure was conducted according to a previously published method (Shan et al., 2021). In brief, samples were treated with ammonium hydroxide, followed by the classic phenol chloroform DNA extraction method. The resulting DNA pellet was air-dried for 5 min, dissolved in 100 μl Tris-Cl (10 mM, pH 8.5) and kept at −20°C.

DNeasy Blood & Tissue Kit was used to extract DNA from *Borrelia* cultures and other bacterial cultures used in this study following the manufacturer’s instructions.

The Invitrogen™ Qubit™ 3 Fluorometer was used to measure the quantity and quality of DNA samples with Qubit™ dsDNA HS and BR Assay Kits following the manufacturer’s instructions. The weight of the *B. miyamotoi* FR64b DNA was converted into copy number according to an online calculator offered by Thermo Fisher (DNA Copy Number and Dilution Calculator).

### Construction of the positive control plasmid DNA

A plasmid carrying the phage *terL* gene fragment from *B. miyamotoi* (named as Bmer-plasmid) was constructed by the Protein Expression Laboratory Service^2^ based on the Vector pLEICS-01. Throughout the PCR setup, 100 copies of the Bmer-plasmid were used as a positive control.

### The Bmer-qPCR

Detailed information regarding the primers and probe used in this study can be found in the approved patent (Ref. P184103.EP.01/T.) (Shan et al., 2018). Briefly, a pair of PCR primers and a probe (the Bmer-qPCR) were designed using the PrimerQuest® Tool to amplify a 120 bp region of the *Borrelia miyamotoi* FR64b *terL* gene (GenBank accession CP004220.1), The primers were F_terBm_:AGCCTACCTAGATCCTGCTTAT and R_terBm_:GGGTCACTTGCTGGTAGTTT. The probe was P_terBm_:AGTGCACTTTGTGTGCTTGAAATGGT. The fluorogenic probe was labelled with 6-carboxyfluorescein (FAM) fluorescent reporter dye at the 5′-end, an internal ZEN™ Quencher and an Iowa Black Fluorescent Quencher (IBFQ) to the 3′ (5’FAM/ZEN/3’IBFQ). Primers and probe of the Ter-qPCR for detecting *B. burgdorferi* s.l. were previously established (Shan et al., 2021). All the primers, probes and PrimeTime Gene Expression Master Mix were supplied by Integrated DNA Technologies (IDT).

### PCR setup

The Bmer-qPCR and Ter-qPCR were conducted according to our established protocol (Shan et al., 2021). Briefly, each primer and probe at a final concentration of 0.5 and 0.25 μM, respectively, were added in a 20 μl final reaction volume containing 10 μl 2X PrimeTime Master Mix, with 4 μl template DNA, and nuclease-free water.

The qPCR result was analysed using FAST7500 software v2.3. For a qPCR result to be valid, the non-template control (NTC) and the extraction control should be negative, whilst positive controls of 100 copies of Bmer-plasmid and Ter-plasmid (Shan et al., 2021), respectively should produce typical qPCR amplification curve with Cq values below 30. When analysing tick pools, positive qPCR results were defined as those with typical PCR curves and Cq ≤ 38. For samples with typical qPCR curves but displaying Cq between 38 and 40, PCR products were gel purified and sequenced (Source Bioscience). All samples were tested in triplicate. Tick pools were scored as positive if at least two PCR repeats were positive in triplicate.

### Analytical specificity and sensitivity

The analytical specificity of the Bmer-qPCR assay was determined using both *in silico* and *in vitro* analyses. *In silico* analysis of the primer and probe set was carried out using BLAST and Primer-BLAST (Ye et al., 2012), and UCSC *In Silico* PCR.^3^ For *in vitro* analysis, the Bmer-qPCR was applied to DNA extracted from a panel of *Borrelia* strains that cause LD and RF (Table 1) and the other non-*Borrelia* strains listed in the section of ‘Bacterial strains’. Additionally, human female and male DNA (Promega, G1521 and G1471) were also tested.

The analytical sensitivity of the Bmer-qPCR was evaluated with five tenfold dilutions of the *B. miyamotoi* FR64b DNA (80,000 to 8 copies), respectively. Each dilution was tested in triplicate to determine the PCR linearity and amplification efficiency. The LOD was then estimated by testing FR64b DNA dilutions from 100 copies to 80, 40, 20, 10, 8, 4, 2 and 1 copies/PCR. Ten replicates were used for each dilution. Probit analysis via the SPSS software was performed to calculate the LOD with 95% probability (Forootan et al., 2017).

### Statistical analysis

GraphPad Prism 8.4.3. was used for statistical analysis. Probit analysis was carried out via the SPSS software suite (IBM SPSS Statistics 25) to estimate the LOD. Linear regressions were used to establish correlations between the serial dilution of FR64b DNA and Cq values.

## Results and discussion

Previously, we conducted a terminase gene-based phylogenetic analysis that revealed *B. miyamotoi*’s distinct clade from LD and RF *Borrelia* species, indicating its importance in the *Borrelia* phylogenetic tree (Shan et al., 2021). The study also suggested that the terminase gene could serve as a potential marker to detect and distinguish *B. miyamotoi* from other *Borrelia* species. Building upon our previous research, we developed a terminase-based PCR (Bmer-qPCR) specifically for detecting *B. miyamotoi*. To investigate the prevalence of *Borrelia* species in UK ticks, it is important to note that *B. miyamotoi* is the only RF *Borrelia* species that can be co-transmitted with LD *Borrelia* species by hard-bodied ticks (Krause et al., 2015). Therefore, we applied both the Ter-qPCR and Bmer-qPCR to the same panel of tick pools collected throughout Great Britain using a citizen science approach to obtain a comprehensive view of the *Borrelia* species distribution. It is important to clarify that our assay is unable to differentiate between the specific Lyme disease species present in a positive tick pool.

### The *terL* gene within *Borrelia miyamotoi* species

The *terL* gene (CP004220.1:11820-13172) was discovered from *B. miyamotoi* FR64b. Blastn search confirmed that its homologues (with E value of 0, 100% Query Cover and > 90% Percent Identity) were widespread within *B. miyamotoi* strains, such as American *B. miyamotoi* strain LB2001, CT13-2396, European *B. miyamotoi* strain NL-1R-1 and Russian *B. miyamotoi* strain Izh-5 (CP024217.1:5875-7227) and *B. miyamotoi* strain Yekat-18 (CP037516.1:247-1598).

The *terL* gene encodes for the terminase large subunit, which is an essential component of the phage packaging system. Phages are viruses that infect bacteria, and they use the host bacterium’s machinery to replicate and package their own genetic material. The terminase is responsible for recognising and cutting the viral DNA into small segments, which are then packaged into new phage particles (Sun et al., 2012).

The discovery of the *terL* gene in *B. miyamotoi* is important because it suggests that this bacterium may have acquired the gene from a phage. This could have important implications for understanding the evolution and pathogenesis of *B. miyamotoi*, as well as for the development of diagnostic tools. Additionally, the *terL* gene could serve as a potential marker for detecting and distinguishing *B. miyamotoi* from other *Borrelia* species.

The discovery of homologues of the *terL* gene in other *B. miyamotoi* strains is significant for our research in several ways. Firstly, it confirms the widespread distribution of the terminase gene within *B. miyamotoi* strains, which supports the use of this gene as a potential marker for detecting and distinguishing *B. miyamotoi* from other *Borrelia* species.

Secondly, it indicates that this gene is likely to be conserved across different geographic regions and ecotypes. This information is useful for future studies on the population genetics and evolutionary history of *B. miyamotoi*, as it suggests that the terminase gene could be used as a tool for investigating the relationships between different *B. miyamotoi* strains. Finally, it highlights the importance of conducting further research on the molecular mechanisms of phages associated with *B. miyamotoi*, particularly with regard to DNA replication and packaging. This information could be used to develop more effective diagnostic tools and therapeutic interventions for *B. miyamotoi* infections.

### Bmer-qPCR analytical specificity, sensitivity and efficiency

The specificity of the primers and probe for Bmer-qPCR was confirmed by Blastn and *In silico*’ PCR.^4^ Positive Bmer-qPCR results were obtained from two strains of *B. miyamotoi*, as listed in Table 1. No positive Bmer-qPCR was observed from any RF-causing *Borrelia* strains or other non-*Borrelia* bacterial strains tested, along with human DNA samples. This demonstrates that the assay is specific for *B. miyamotoi* and does not cross-react with other *Borrelia* or non-*Borrelia* bacterial strains or human DNA samples.

**Figure 1:**
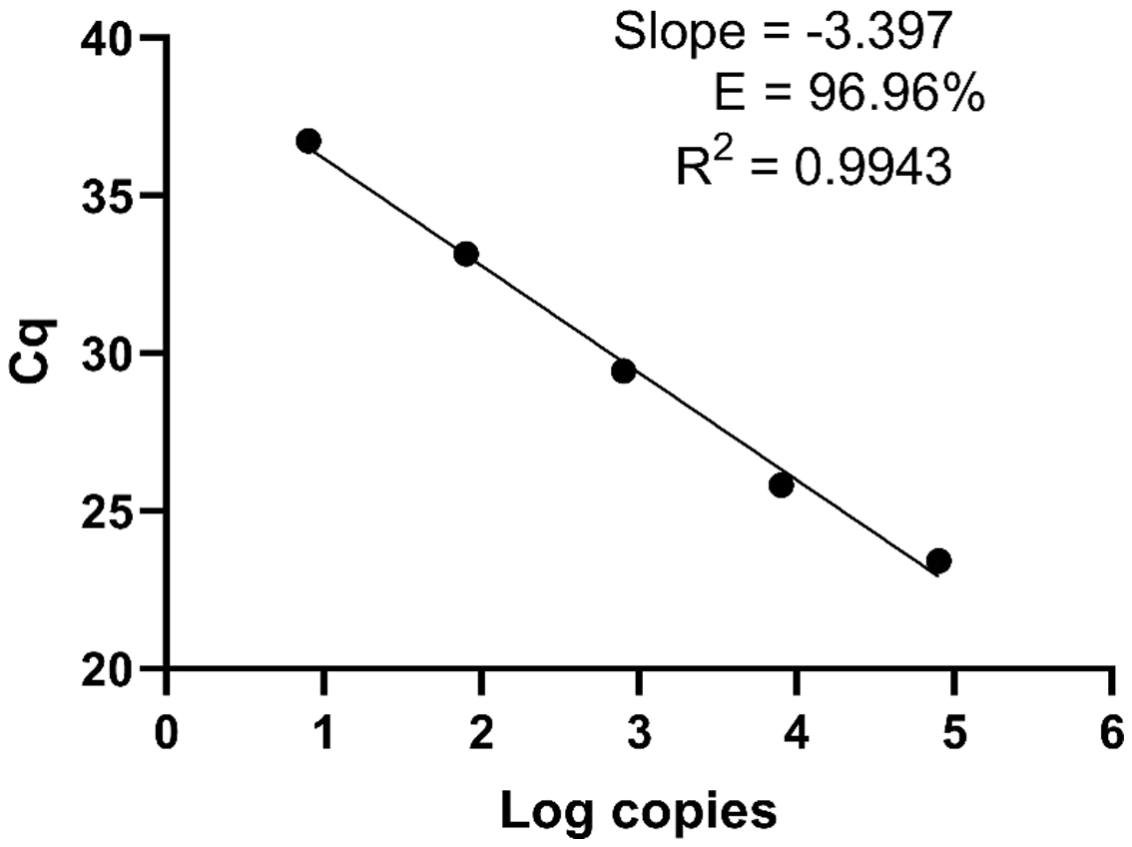
The Bmer-qPCR against 10-fold serial dilutions of the *B. miyamotoi* FR64b DNA to measure the PCR efficiency. Cq values were plotted against the log values of serial dilutions of the FR64b DNA. Simple regression analysis was carried out using the Graphpad Prism 8.4.3 software. The slope, coefficient of correlation (*R*^2^) and efficiency of the reaction (E) are shown. Each dot represents the average value from triplicate amplifications, along with the SD.

To determine the qPCR efficiency, the Bmer-qPCR assay was conducted in 10-fold dilutions of *B. miyamotoi* FR64b DNA. We observed a strong linear relationship between the concentration of the DNA and Cq (*R*^2^ = 0.9943) with an amplification efficiency of 96.96% (Figure 1). This demonstrates the high efficiency of the Bmer-qPCR. A high PCR efficiency coupled with a strong linearity from serial dilution experiment confirms that the Bmer-qPCR is reliable.

In this study, we defined the analytical limit of detection (LOD) as the lowest concentration where at least 95% of the technical replicates were positive in the Bmer-qPCR (Wei et al., 2016). To estimate the LOD, diluted *B. miyamotoi* FR64b DNA was used. The FR64b DNA was diluted from 100 copies to 80, 40, 20, 10, 8, 4, 2 and 1 copies/PCR. Ten replicates were used for each dilution. Positive qPCR has typical PCR amplification curve with a Cq ≤ 38. The proportion of Bmer-qPCR positive amongst replicates was directly correlated with the number of copies per reaction (Table 2). For example, one copy of the FR64b DNA led to two positives out of 10 replicates, whilst 4 and 8 copies generated 9 and 10 positives out of 10 replicates, respectively. Probit analysis via the SPSS package with 95% probability was used to estimate the LOD and was found to be 10 copies per PCR (Pavšič et al., 2016). This means that the assay can detect as low as 10 copies of *B. miyamotoi* DNA per PCR reaction with a 95% confidence interval.

**Table 2:** Estimation of LOD of the Bmer-qPCR using diluted *B. miyamotoi* FR64b DNA.

Copy number/PCR	Number of replicates	Number of positive replicates	% of positive
1	10	2	20
2	10	5	50
4	10	9	90
8	10	10	100
10	10	10	100
20	10	10	100
40	10	10	100
80	10	10	100
100	10	10	100

Overall, the validation results of the Bmer-qPCR assay for the detection of *B. miyamotoi* DNA suggest that the Bmer-qPCR assay is a reliable and sensitive tool for the detection of *B. miyamotoi* DNA.

### Tick collection

An initial public ‘recruitment’ campaign was carried out at the British Shooting Show 2016 followed by communications with The British Deer Society and the National Gamekeepers Organisation. The Ministry of Defence Sustainability Magazine, ‘Sanctuary’, promoted Leicester’s tick collection campaign and published Leicester’s article named ‘COMBATTING LYME DISEASE: PHAGES THE SHARPSHOOTERS OF ENEMY BACTERIA’.

A tick collection package was designed to be easily understandable and postable via the UK Postal Service’s First Class Mail Letters. Each tick package includes:

1. A tick remover
2. Several Falcon™ 15 ml tubes and paper towels
3. Sealable plastic bags to hold the Falcon™ tubes
4. An instruction letter (Supplementary Information 1) highlighting the need to record geographical locations, date and tick origin (deer, dog, etc.)
5. Prepaid postage sealable return padded envelopes.

Ticks collected from the same location on the same date and from the same origin were combined into a tick pool. It is worth mentioning the importance of a user-friendly tick collection package that includes all the necessary equipment and instructions for collecting and submitting tick samples.

The campaign’s success highlights the crucial role in the collaboration with various organisations and the promotion through different channels. The power of citizen science was demonstrated in tick sampling from a large geographical area. The campaign for a citizen scientist-based approach launched in April 2016. Since July 2016, we started receiving requests for our tick kits. We sent out 235 tick kits by post. Each kit has space for 1–10 tick collection tubes depending on the request. The data presented in this study represents tick collection from March to August 2017 (the tick season). As of 30 August 2017, 211 tick kits had been returned. Amongst these, 153 tick kits from 66 geographical locations (Supplementary Table 1) recorded accurate locations with 838 correctly identified adult ticks (multiple tick kits from the same location in different date and/or tick origins were received). Ticks from the same tick kit (representing ticks collected from the same location, on the same date and from the same origin as illustrated in Supplementary Table 1) were pooled together to form 153 tick pools, representing 66 geographical locations (6 regions in England and 5 regions in Scotland). The collected data could be valuable in studying the distribution of ticks and the prevalence of LD in the UK, ultimately leading to better management and prevention strategies.

### Prevalence of *Borrelia burgdorferi s.l.* and *Borrelia miyamotoi* in tick pools: The spread of *Borrelia miyamotoi*

This study is part of an effort to understand the transmission and spread of two *Borrelia* species, *B. burgdorferi s.l.* and *B. miyamotoi*, in ticks. To generate large-scale, nationwide data, we focused on three key components: (1) The development of accurate PCR tests for the detection of *Borrelia* species; (2) The establishment of a volunteer network using a citizen science approach and (3) The application of *Borrelia-*specific PCRs to a large collection of tick pools.

The estimation of *Borrelia* carriage rate can be based on either single ticks or tick pools. To calculate the *Borrelia* carriage rate based on single ticks, each tick is individually tested for the presence of *Borrelia*. Testing each tick individually provides accurate information about the prevalence of *Borrelia* in ticks, as it can identify which individual ticks are infected. However, it can be more time-consuming and expensive to test each tick individually (Raulf et al., 2018). To calculate the *Borrelia* carriage rate based on tick pools, a group of ticks is combined into a single sample, or pool, which is then tested for the presence of *Borrelia*. This method can increase sample size, which is particularly important when studying rare events, such as the presence of *Borrelia* infections in ticks. Pooling ticks can be a cost-effective method compared to analysing each tick individually. By pooling ticks from the same location/time, the prevalence of *Borrelia* infections in ticks can be determined for that specific location/time. This information can be used to develop targeted interventions to reduce the risk of LD in that area.

TBDs are a major public health concern worldwide. Studies have shown that the prevalence of *B. burgdorferi* s.l. and *B. miyamotoi* in ticks varies depending on geographic location.

For instance, a single tick study conducted by Xu et al. in 2020 revealed a contrasting rates of *B. burgdorferi s.l.* carriage in ticks. In the southern US, 2–8% of ticks showed *Borrelia* carriage, compared to infection rates averaging 50% in New England (Xu et al., 2020).

Similarly, single tick testing in central Britain revealed that the prevalence of *B. burgdorferi* s.l. ranges from <1.0 to >7.5% (Bettridge et al., 2013).

Single tick testing also showed that the *B. miyamotoi* carriage rate in ticks is generally much lower than that of *B. burgdorferi s.l.* In the US, the estimated infection rate of *B. miyamotoi* in ticks was 0.5 to 3.2% during 2013–2019 (Xu et al., 2021), whilst in southern England and northern Italy, the prevalence of *B. miyamotoi* was 0.3 and 0.74%, respectively (Hansford et al., 2015; Ravagnan et al., 2018). A survey in France, Denmark and the Netherlands estimated a prevalence of between 0.17 to 3.36%, whilst a study in northeast Inner Mongolia found that 1.3% of ticks were B. miyamotoi-positive (Crowder et al., 2014; Gao et al., 2021).

Studying tick pools can also provide useful information. For example, Padgett et al. (2014) studied tick pools to compare the prevalence of *B. miyamotoi* and *B. burgdorferi* s.l. in the western black-legged tick, *Ixodes pacificus*, across California. The results showed significant variation in the prevalence of both *Borrelia* species between locations, indicating that control strategies should consider local prevalence patterns (Padgett et al., 2014). This suggests that the risk of exposure to *B. miyamotoi* and *B. burgdorferi* s.s. may vary depending on location, and that control strategies should take into account local prevalence patterns.

Studying tick pools also helped provide the first evidence of the presence of Borrelia miyamotoi in Thailand (Takhampunya et al., 2023). The study identified B. miyamotoi in *Ixodes granulatus* collected from a wildlife sanctuary in Thailand. The results were confirmed by molecular techniques, which also revealed that the Thai strains of *B. miyamotoi* were more closely related to strains found in Japan and China than to those found in Europe and North America. The authors suggest that more research is needed to understand the prevalence and distribution of *B. miyamotoi* in Thailand and other countries in the region, as well as its potential impact on public health.

Our study supports the findings of Padgett et al. regarding the relationship between the prevalence of *B. burgdorferi* s.l./*B. miyamotoi* in ticks and their geographic location. We found that *B. miyamotoi* coexists with *B. burgdorferi* s.l. in the same area, and that the prevalence of both species varies by location (Supplementary Table 1). Tick pools in England had a higher average prevalence of *B. miyamotoi* (6.9%) than those in Scotland (4.5%), whilst the prevalence of *B. burgdorferi* s.l. was higher in Scottish tick pools compared to those in England.

To identify patterns in the spread of *Borrelia*, we compared levels of *Borrelia* carriage in different regions of England and Scotland. As shown in Supplementary Table 1, we observed a complementary pattern of high *B. burgdorferi* s.l. and low *B. miyamotoi* in the north, and high *B. miyamotoi* and low *B. burgdorferi* s.l. in the south. For example, tick pools collected from South East England exhibited a high rate of *B. burgdorferi* s.l. carriage (71.4%, similar to the overall average as shown in Supplementary Table 1) and a lower rate of *B. miyamotoi* carriage (14.3%), whilst tick pools from the Highlands of Scotland showed an above-average *B. burgdorferi* s.l. carriage rate (91.7%) and a low rate of *B. miyamotoi* carriage (0%). This interesting pattern of *Borrelia* in ticks has implications for the pathogenesis of LD. The underlying reasons for this north–south distribution of *Borrelia* highlight the value of a citizen science approach and the need for larger-scale tick research.

In conclusion, TBDs are complex and multifactorial, with the prevalence of *Borrelia* species varying by location. Studying tick pools can provide valuable information about local prevalence patterns, which can inform control strategies and public health interventions.

### Ticks from deer and dogs

A total of 55 tick pools were prepared from deer, with 72.7% testing positive for *B. burgdorferi* s.l. and 5.5% for *B. miyamotoi*. These rates are similar to the overall average *Borrelia* carriage rates of 71.9% for *B. burgdorferi* s.l. and 5.9% for *B. miyamotoi* (Supplementary Table 1). Although deer are well-known as major tick hosts, they do not carry or transmit *Borrelia* bacteria themselves (Mysterud et al., 2016). Consequently, *Borrelia*-positive ticks found on deer must have acquired the bacteria from previous hosts. The correlation between increasing deer densities and increasing tick populations has been reported before based on the fact that deer are hotbeds for tick reproduction (Kilpatrick et al., 2014). However, whether increased number of deer would lead to increased *Borrelia*-infected ticks, hence increased human infection rate remains to be understood and investigated (Gandy et al., 2021).

Fifty-one tick pools were prepared from dogs. Amongst these dog tick pools, 67.7 and 3.9% tested positive for *B. burgdorferi* s.l. and *B. miyamotoi*, respectively. The fact that *Borrelia*-positive ticks are readily found from domestic dogs highlights the need for accurate understanding and monitoring of tick prevalence patterns. A study carried out in 2009 demonstrated that 810 out of a total of 3,534 dogs were found to be carrying at least one tick(Smith et al., 2011). Our study indicated that it might be common for dog ticks to be positive to LD-causing bacteria and RF-causing bacteria. It is probable that the combination of high rates of *Borrelia* carriage in dog ticks and low awareness of *Borrelia* association with domestic dogs could result in a situation with high potential for a pandemic. More research on the spread of *Borrelia* via tick-domestic animal route, and the potential impact of infected ticks on human beings via the domestic animal route are needed.

### Citizen science

The citizen scientists-led method offers a large-scale sampling which would not otherwise be possible, but has several limitations. First, analysis of tick pools provides no information on associations between *Borrelia* species and specific ticks. Single tick analysis will be required to investigate the carriage of *Borrelia* in individual ticks. Second, we pooled ticks from the same location, but did not exclude any ticks visibly blood engorged. Therefore, we could not rule out the possibility of the previous blood meal influencing the *Borrelia* carriage in each tick (Rynkiewicz et al., 2015). Single tick analysis will address this limitation. Finally, our citizen science-based method, whilst allowing the collection of large numbers of ticks, did not oversee a standardised collection procedure and an unbiased geographic sampling. For example, no ticks were collected from Wales and Midland of England due to a lack of citizen scientists. Limitations in our current citizen science design may be addressed using smaller cohorts of ticks collected by trained researchers.

The last decade has seen an increase in TBDs worldwide. Here, we demonstrated the potential of phage-based PCR methods coupled with citizen science to support large-scale, sample analysis across broad geographic areas. Throughout this project, we collected ticks from across Great Britain. Although our initial focus was on *Borrelia* species, our PCR methods are applicable to other tick-borne pathogens. We anticipate a follow-up project focusing on single tick level, which would engage with communities at risk from tick bite to provide real-time information on the spread of tick-borne pathogens and inform relevant agencies to develop corresponding control measures.

## Data availability statement

The original contributions presented in the study are included in the article/Supplementary material, further inquiries can be directed to the corresponding author.

## Author contributions

JS and YJ contributed equally to this work and co-wrote the manuscript. JS and MC co-conceived the initial idea. JS expanded the initial idea into a coherent scientific project, managed the citizen science project, designed primers and probes, and performed in-depth data analysis. YJ was responsible for experimentation and optimisation, data collection, and initial data interpretation. PH helped to coordinate the tick collection using the citizen science approach. MC proofread the manuscript. LT proofread the manuscript and provided valuable advice on *B. miyamotoi* clinical diagnosis and treatment. All authors contributed to the article and approved the submitted version.

## Funding

The main funding received towards the study from the Phelix Research and Development (Phelix R&D, 37 Langton Street, SW10 0JL London, UK, the Charity Number 1156666), and the University of Leicester Drug Discovery and Diagnostics (LD3) fund 2022.

## Conflict of interest

LT is a physician who volunteers for Phelix Research and Development, a charitable organization. The other authors confirm that this research was conducted without any commercial or financial relationships that could potentially be considered conflicts of interest.

## Publisher’s note

All claims expressed in this article are solely those of the authors and do not necessarily represent those of their affiliated organizations, or those of the publisher, the editors and the reviewers. Any product that may be evaluated in this article, or claim that may be made by its manufacturer, is not guaranteed or endorsed by the publisher.

## Footnotes

^1^ http://www.bristoluniversitytickid.uk/

^2^ https://le.ac.uk/mcb/facilities-and-technologies/protex

^3^ https://genome.ucsc.edu/cgi-bin/hgPcr

^4^ http://insilico.ehu.es/PCR/

## Supplementary material

SUPPLEMENTARY TABLE 1 Prevalence of *B. burgdorferi s.l.* and *B. miyamotoi* in tick pools across Great Britain.
