# Supplementary material for: Combining citizen science and molecular diagnostic methods to investigate the prevalence of Borrelia burgdorferi s.l. and Borrelia miyamotoi in tick pools across Great Britain
Source: Front Microbiol. 2023 Apr 26;14:1126498. doi: 10.3389/fmicb.2023.1126498 (PMC10169747; doi:10.3389/fmicb.2023.1126498)
Supplement: Supplementary file 2 [file Data_Sheet_1.DOCX]

**Participant Instructions For Sending Ticks In Post**

- The kit contains all the items needed to remove and send ticks
- Number tubes (1,2…) and provide brief description:

Animal ticks came from

Date ticks were collected

Geographical location, such as postal code ticks were collected

Any other useful information about ticks collected

- More than 1 tick can be put in 1 tube if collected from the same animal and location
- Unscrew and remove caps on tubes, put ticks in tubes, replace caps and tighten
- Wrap the tubes in paper towelling, place in grip seal bag and seal bag (the grip seal pulls apart and re-seals when pressed together)
- Put grip seal bag containing tubes and ticks in white pre-paid protective return bag addressed to Jinyu Shan & Martha Clokie and seal bag by removing protective paper strip and folding down self-adhesive flap
- Send bag in post
- PS. Ticks can be stored in a refrigerator (4°C) for up to 2 months and sent together if collect at different times

**Thank you for participating in our research work**


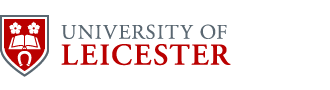


Ref: PTH 11/7/16
